# POLAR: prediction of prolonged mechanical ventilation in patients with myasthenic crisis

**DOI:** 10.1007/s00415-024-12208-4

**Published:** 2024-02-08

**Authors:** Dominik Heider, Henning Stetefeld, Andreas Meisel, Julian Bösel, Marie Artho, Ralf Linker, Klemens Angstwurm, Bernhard Neumann, The German Myasthenic Crisis Study Group, Bernhard Neumann, Kornelius Fuchs, Amelie Vidal, Lee De-Hyung, Siegfried Kohler, Philipp Mergenthaler, Izabela Brachaczek, Jana Maidhof, Arno Wenke, Christian Dohmen, Silvia Schönenberger, Stefan Gerner, Hagen Huttner, Manuel Hagen, Hauke Schneider, Heinz Reichmann, Jan Rahmig, Eik Schimmel, Wolf Niesen, Hannah Fuhrerer, Christiane Schneider-Gold, Ingo Kleiter, Christine Fahrendorf, Andrea Thieme, Andreas Steinbrecher, Juliane Dunkel, Christian Roth, Anke Alberty, Jan Zinke

**Affiliations:** 1 Department of Machine Learning for Medical Data, Institute for Computer Science, Heinrich-Heine-Universität Düsseldorf, Düsseldorf, Germany; 2 Department of Data Science in Biomedicine, Faculty of Mathematics and Computer Science, University of Marburg, Marburg, Germany; 3 Department of Neurology, Faculty of Medicine, University Hospital Cologne, University of Cologne, Cologne, Germany; 4 Department of Neurology With Experimental Neurology, Neuroscience Clinical Research Center, Charité, Universitätsmedizin Berlin, Berlin, Germany; 5 Department of Neurology, Heidelberg University Hospital, Heidelberg, Germany; 6 Department of Neurology, Johns Hopkins University Hospital, Baltimore, MD USA; 7 Department of Neurology, University of Regensburg, Bezirksklinikum, Regensburg, Germany; 8 Department of Neurology, Donau-Isar-Klinikum Deggendorf, Perlasberger Straße 41, 94469 Deggendorf, Germany

Dear Sirs,

Myasthenia gravis (MG) is a B-cell-mediated autoimmune disease and the most frequent disorder of the neuromuscular junction. Characteristic symptoms are muscle weakness, dysphagia, diplopia and fatigue that typically increase throughout continuous muscular activity [1, 2].

Diagnosis of MG typically involves clinical evaluation and diagnostic testing such as low-frequency repetitive nerve stimulation, blood tests to detect specific antibodies, and imaging studies of the thymus gland, which is often involved in the development of the disease. Treatment for MG includes the use of medications to improve muscle function and suppress the autoimmune response. Cholinesterase inhibitors and immunosuppressive medications are commonly used to manage MG symptoms. Living with MG can be challenging and within 2 years of the initial diagnosis, 15–20% of MG patients develop a potentially life-threatening myasthenic crisis (MC) [3]. MC has been defined as respiratory failure requiring mechanical ventilation (MV) due to respiratory muscle weakness or bulbar dysfunction. The mortality, even in our times, is around 12% and prolonged MV (defined as more than 15 days) occurs in 44.7% of the MG patients [4]. Analysis of invasive ventilated patients showed that in eligible patients, early tracheostomy can be associated with a shorter mechanical ventilation duration and a shorter stay in the intensive care unit [4, 5]. Weaning and extubation failure occur even in our times frequently [6]. The prediction of prolonged MV is still very challenging, since many factors such as precondition of the patient, treatment and characteristic of the MG play an important role. No prediction model has been developed to date due to the absence of large cohorts of patients with MC requiring MV.

In order to improve the individual treatment of the patients with MC and to estimate the risks for prolonged mechanical ventilation, we developed POLAR, a prediction model and an easy-to-use web-app for physicians.

We used the largest multicenter cohort available [4] that includes 149 features of 195 patients with invasive ventilation, where the exact number of days of mechanical ventilation is known. The features include baseline demographics, clinical features, medication, comorbidities, characteristics of the MG, treatment regimens during MC, complications of the MC, and outcome parameters like death and days of mechanical ventilation as published before [4, 5]. The main outcome parameter of interest in this analysis was a prolonged mechanical ventilation, defined as MV for more than 15 days.

First, we manually selected 11 features from our dataset. These features are complete (i.e., no missing values) and were selected based on expert experience and previous analyses [4, 5]. Next, we used the EFS (Ensemble Feature Selection) package in R for feature selection [7, 8]. EFS is based on multiple feature selection methods and combines their normalized outputs to quantitative ensemble importance, thereby reducing the individual biases of the methods and providing an unbiased biomarker discovery.

We used a logistic regression (LR) model. After training, it can be employed to predict the probability of the binary outcome for new input data. The logistic regression model uses the following equation:

$$\begin{aligned} x_{i}^{T} \beta & = 1.8267 - 0.0044 \times {\text{age}} - 0.3814 \times {\text{comorbidities}} - 17.4621 \times {\text{MGFA}}_{{{\text{IVb}}}} \\ & - 1.0911 \times {\text{late}}_{{{\text{onset}}}} + 0.5329 \times {\text{FM}}/{\text{MGFA}}_{{{\text{II}}}} - 1.2921 \times {\text{pneumonia}} \\ & - 0.6845 \times {\text{delirium}} \vee {\text{psychosis}} - 2.005 \times {\text{CPR}} + 0.4586 \times \hom e \\ \end{aligned}$$

where FM/MGFA_II_ represents patients with first manifestation of MG during MC or patients with a MGFA score of II or less before MC; CPR represents patients requiring cardio-pulmonal resuscitation during MC; home represents patients who were living at home independent of help before MC.

For evaluation of the LR, we used repeated 10-times tenfold cross-validation. For calibration of the model, we employed GUESS from the R package CalibratR [9].

As an evaluation metric, we employed the Receiver Operating Characteristics curve (ROC) and the corresponding Area Under the Curve (AUC) using the pROC [10] package in R. A one-sided Student’s *t* test was used for statistical analyses.

We made the LR model available as a web-application using Shiny v.1.7. Shiny is a framework for R that facilitates the development of interactive web applications. Shiny leverages a feature known as reactivity to support interactivity in applications. This enables users to modify input values and have the displayed R objects automatically update in response.

EFS identified nine biomarkers (see Fig. 1) above our threshold of 0.4, namely the age of the patient, the prevalence of at least three chronic comorbidities affecting different organ systems (lung, heart, kidney, diabetes (pancreas) and/or a tumor disease [except thymoma]), late-onset MG, a high Myasthenia Gravis Foundation of America (MGFA) class (IVb) before crisis, implying a severe general course of MG, and delirium, pneumonia or cardio-pulmonary resuscitation during MC can be used to predict prolonged mechanical ventilation. A first manifestation through MC, as well as low MGFA class before MC or whether the patients were independent before MC were markers for a reduced likelihood for prolonged mechanical ventilation. Being a smoker before MC or having a sepsis during MC was no biomarkers with relevant impact (Fig. 1) for our prediction model.

**Fig. 1:**
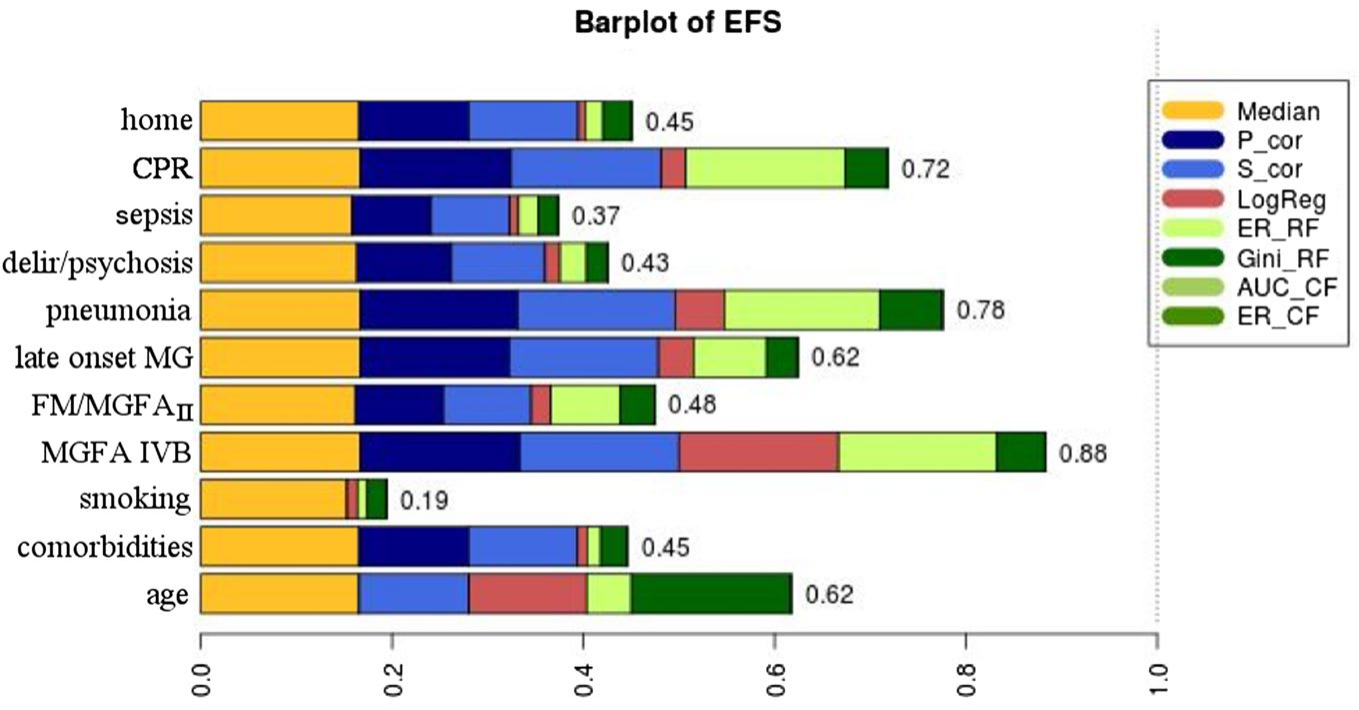
EFS bar plot. EFS removed two features from our selection, i.e., the importance is below the threshold (0.4). home: patients who lived at home independently of help before MC; CPR: patients requiring cardio-pulmonal resuscitation during MC; FM/MGFA_II_: patients with first manifestation of MG during MC or patients with a MGFA score of II or less before MC; MGFA IVB: patients with an MGFA score of IVB before MC; comorbidities: patients with at least three comorbidities before MC. Median: Median filter; P_cor: Pearson correlation filter; S_cor: Spearman correlation filter; LogReg: logistic regression wrapper; ER_RF: random forests importance analysis based on error rate; Gini_RF: random forests importance analysis based on Gini impurity; AUC_CF: conditional forests importance analysis based on AUC; ER_CF: conditional forests importance analysis based on error rate

The identified features were used to train a logistic regression model, as described earlier and all nine biomarkers with relevant impact according to the feature selection were included (see methods section).

Our prediction model is able to accurately (AUC = 0.78, *p* < 0.00001) (Fig. 2) predict the likelihood (in percentage) whether a prolonged mechanical ventilation is necessary for more than 15 days in a given MG patient, based on our cohort. The prediction model is available (in English and German) as a R/Shiny web-app at http://POLAR.heiderlab.de.

**Fig. 2:**
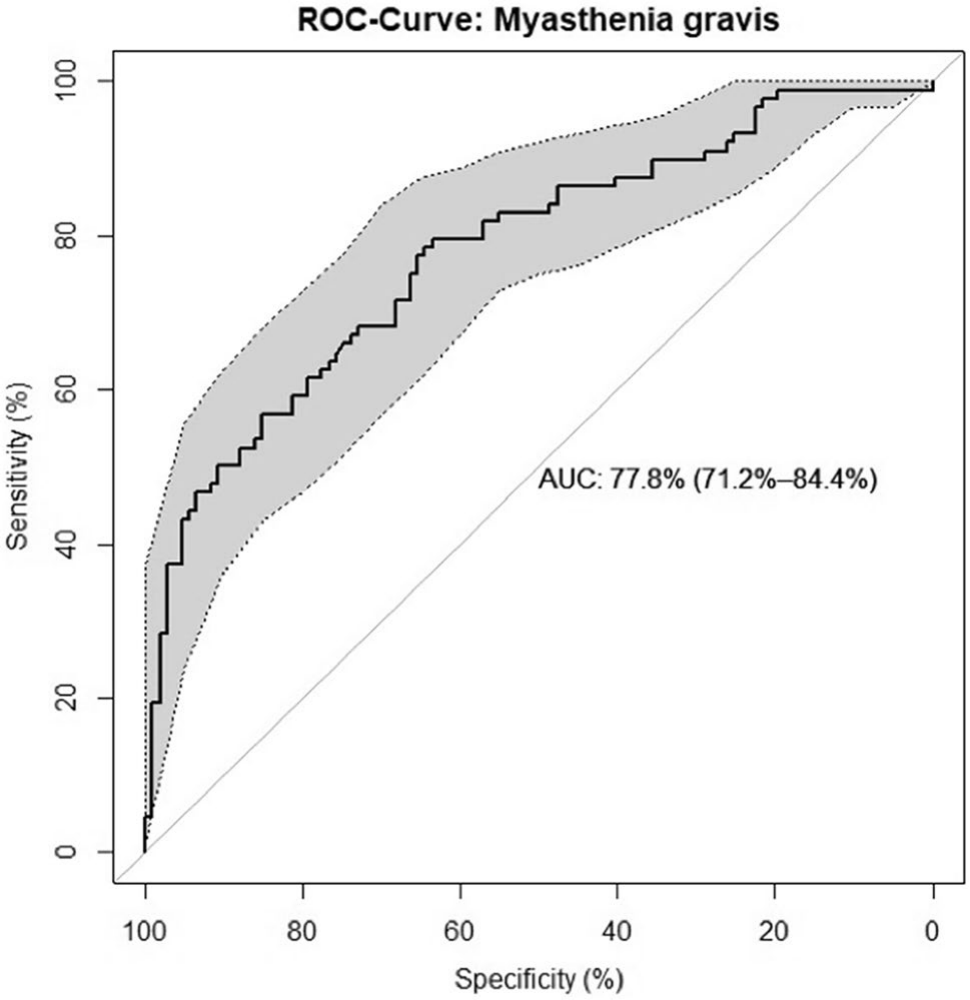
ROC curve of the logistic regression model. The confidence interval of the ROC curve is shown in gray (*p* < 0.00001). Figure is drawn with pROC

POLAR is a prediction model based on data of the largest retrospective cohort of ventilated MC patients [4]. The model comprises six biomarkers associated with prolonged ventilation including age, the prevalence of at least three chronic comorbidities, late-onset MG, a high MGFA class before crisis, delirium, pneumonia or cardio-pulmonary resuscitation during MC. Moreover, there were three biomarkers associated with a reduced ventilation including a first manifestation with MC, as well as low MGFA class and high independence before MC. Thus, our prediction model is able to predict the likelihood of prolonged MV (> 15 days), based on our cohort.

Like in other cohorts of MC patients, higher age is a reasonable parameter for prolonged MV or poor outcome and not specific for MG [11–13]. In addition, we found that late-onset MG is a risk factor for prolonged MV, probably overlapping with higher age as a factor, since most of our patients belonged to the late-onset group (80%). We found that a higher MGFA score, before MC increased the likelihood of prolonged mechanical ventilation in our cohort [4]. Other groups found similar results when analyzing Myasthenia Gravis Activities of Daily Living (MG-ADL) scales [12]. Lui et al. also confirmed that a higher number of comorbidities were associated with poor outcome which is well in line with our observation [12]. Another biomarker in our model was pneumonia, which was identified as factor associated with prolonged MV before [14], conforming our results. Sepsis during MC was associated with prolonged MV in another cohort [15]. Yet, in our cohort, the effect did not show a relevant impact in the feature selection and, therefore, did not make it into our prediction model. This surprising result could be explained by the fact, that sepsis was the main reason for death in our cohort and 57% of these patients died within 15 days and, therefore, did not belong to the prolonged MV group in our analysis. Most of the complications included in our score, such as delirium and pneumonia and, in some cases, even cardio-pulmonary resuscitation, occurred within the first 5 days of treatment at intensive care unit (ICU). Therefore, it is important to note that the score has a higher reliability between days 5 and 6 of treatment that in the first 48 h at ICU.

In this manuscript, we present for the first time a web-based prediction model to estimate prolonged mechanical ventilation in myasthenic crisis. POLAR can help to estimate prolonged mechanical ventilation and may enable an optimized personalized treatment, including decision making on intensive care unit, of patients with MC. However, our prediction model needs further validation in prospective cohorts and is, at the moment, only a first step towards a reliable prediction model for prolonged mechanical ventilation in myasthenic crisis. Nevertheless, POLAR can be seen as a good starting point for making probabilistic estimates for prolonged mechanical ventilation, which does not only help patients and their families, but also to improve medical care.

## Data Availability

Anonymized data will be made available upon reasonable request.
